# Yeast complementation assays provide limited information on functional features of K^+^ channels

**DOI:** 10.1016/j.bpr.2025.100206

**Published:** 2025-03-13

**Authors:** Kerri Kukovetz, Matea Cartolano, Manuela Gebhardt, Lars E. Schumann, Stefan M. Kast, Anna Moroni, Gerhard Thiel, Oliver Rauh

**Affiliations:** 1 Department of Biology, TU Darmstadt, Darmstadt, Germany; 2 Department of Chemistry and Chemical Biology, TU Dortmund University, Dortmund, Germany; 3 Department of Biosciences and CNR IBF-Mi, Università degli Studi di Milano, Milano, Italy; 4 Institute for Functional Gene Analytics, Department of Natural Sciences, Bonn-Rhein-Sieg University of Applied Sciences, Rheinbach, Germany

## Abstract

We investigate to what extent yeast complementation assays, which in principle can provide large amounts of training data for machine-learning models, yield quantitative correlations between growth rescue and single-channel recordings. If this were the case, yeast complementation results could be used as surrogate data for machine-learning-based channel design. Therefore, we mutated position L94 at the cavity entry of the model K^+^ channel Kcv_PBCV1_ to all proteinogenic amino acids. The function of the wild-type channel and its mutants was investigated by reconstituting them in planar lipid bilayers and by their ability to rescue the growth of a yeast strain deficient in K^+^ uptake. The single-channel data show a distinct effect of mutations in this critical position on unitary conductance and open probability, with no apparent causal relationship between the two functional parameters. We also found that even conservative amino acid replacements can alter the unitary conductance and/or open probability and that most functional changes show no systematic relationship with the physicochemical nature of the amino acids. This emphasizes that the functional influence of an amino acid on channel function cannot be reduced to a single chemical property. Mutual comparison of single-channel data and yeast complementation results exhibit only a partial correlation between their electrical parameters and their potency of rescuing growth. Hence, complementation data alone are not sufficient for enabling functional channel design; they need to be complemented by additional parameters such as the number of channels in the plasma membrane.

## Why it matters

Designing K^+^ channels with new features by machine-learning methods requires large training data sets of variants with confirmed and quantified activity. Here, we examine whether the growth rescue of a yeast mutant by a K^+^ channel of interest offers such a desired high-throughput assay for channel function. We mutated one site in a model K^+^ channel into all proteinogenic amino acids and examined the functional impact by single-channel recordings and yeast complementation. We find that the latter indirect approach provides only a partial correlation between the electrical parameters of the channel mutants and their potency of rescuing growth, suggesting that additional parameters are required for complementing the information on channel function from yeast growth rescue.

## Introduction

By accumulating potassium in the cytosol, cells of baker’s yeast (*Saccharomyces cerevisiae*) generate an osmotically driven influx of water, which in turn builds up an internal pressure for cell growth. When growing in medium with low-millimolar or even sub-millimolar K^+^ concentrations, the energetic uphill transport of K^+^ into the cells is achieved by the concerted action of a H^+^ ATPase and two K^+^ selective transporters (1). One of these transporters, Trk1p, is essential for growth in micromolar K^+^ while the second one, Trk2p, kicks in at low-millimolar K^+^ concentrations (2,3,4). The proton pump hyperpolarizes in this scenario the plasma membrane to values well negative of −100 mV (1). This creates, even at very low K^+^ concentrations in the external solution, together with a favorable H^+^ concentration gradient, an electrochemical gradient for symport of K^+^ with H^+^ into the cells.

Because of this crucial role of the transporters in K^+^ import and survival, their double knockout prevents growth of the trk1Δtrk2Δ yeast mutants in low-K^+^ medium. Survival of this mutant is only possible in medium with ≥50 mM K^+^ (5), since the remaining outward rectifying TOK1 channel is unable to substitute the function of Trk1p and Trk2p for conducting K^+^ influx (6,7). This demand for high concentrations of K^+^ can be overcome by heterologous expression of functional K^+^ channels (6). When these channels are inserted into the plasma membrane of the yeast mutants and are conductive at hyperpolarized voltages, they mediate a K^+^ influx into cells, which promotes growth even in media with low K^+^. The respective trk1Δtrk2Δ yeast mutants have already been successfully used in the past in such complementation assays to examine the function of many K^+^ channels from various sources, including prokaryotes and viruses as well as eukaryotes with mammalian channels (8). The simplicity of these growth assays offers a promising experimental tool for fast screening and selection in a medium-throughput manner of mutant libraries of natural (9,10) and synthetic K^+^ channels (11). This also includes the possibility of detecting functional changes of K^+^ channel activity as a result of point mutations (12). Also, the efficiency of channel blockers and/or modulators can be tested with this assay (8,13). Furthermore, the unique and efficient mechanism of DNA recombination in *S. cerevisiae* can be employed in combination with this growth rescue system as a powerful platform for genetic manipulations of channel proteins ranging from *in vivo* cloning to a steering of molecular diversity (11,14).

Here, we address the question as to what extent the growth phenotype of yeast cells in a complementation assay can be interpreted with respect to basic functional variables such as open probability and/or unitary conductance of the K^+^ channel employed for growth rescue. This kind of information could be very suitable for future applications of machine-learning approaches in the field of ion channels (15,16), as the yeast complementation approach allows in principle for rapid generation of large data sets providing quantitative surrogate training data for these algorithms in the case that the yeast data correlate with structure and function. Hence, they could replace the comparably slower and much more expensive classical electrophysiological characterization of channel mutants.

**Figure 1:**
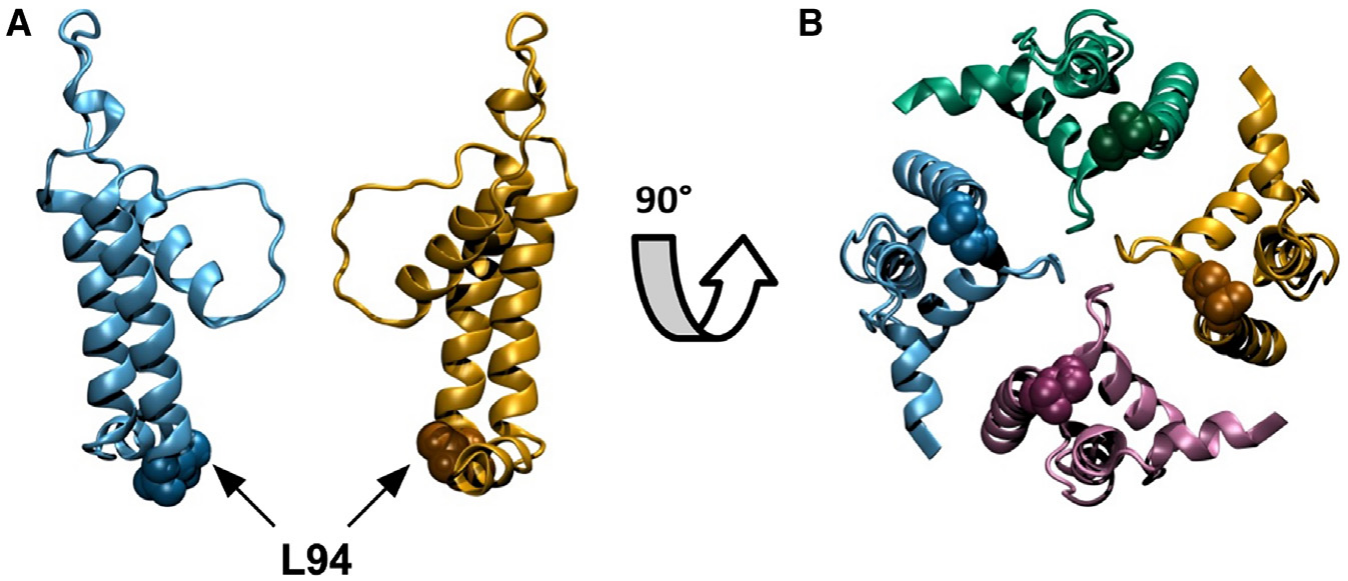
Computationally modeled atomistic representation of the Kcv_PBCV1_ WT structure (L.E.S., unpublished data). (*A*) Schematic cross section showing two out of four monomers in cartoon representation with residue L94 shown in van der Waals representation. Van der Waals spheres have been enlarged by a factor of 1.3 for clarity. (*B*) View of all four monomers after rotation of (*A*) by 90°. The structure shown is a representative structure (10,19) of a proposed active-channel conformation based on molecular dynamics simulations originating from a homology model with template structure NaK2K (20).

In the past, we have used yeast complementation assays in combination with alanine scanning to examine structure/function correlates in the small viral K^+^ channel Kcv_PBCV1_ (Fig. 1). These data showed that substitutions of amino acids in the two transmembrane domains of this channel by alanine generated diverse growth phenotypes, ranging from no to robust rescue of cell growth (9). The different rescue efficiencies were interpreted in combination with electrophysiological recordings as an indication for amino acid positions that are functionally inert against replacement by alanine (= robust rescue) or sensitive to such a mutation (= low or no rescue) (9). One of the amino acid substitutions, which significantly reduced yeast growth in this complementation assay, was the mutation L94A, i.e., a mutation of the last amino acid of the second transmembrane domain (Fig. 1). From experimental and computational simulation studies, it was already anticipated that this position at the entry to the cavity might be crucial for channel gating (10,17,18). To better interpret the information from such complementation assays in the context of structure/function correlates of the channel, we replaced in the present study L94 by all other natural amino acids. The function of these mutant channels was therefore examined both in a yeast complementation assay and by single-channel recordings in planar lipid bilayers. As will be shown, a mutual comparison between the degree of growth rescue and single-channel data from all mutants reveals that the yeast growth phenotypes do only partially reflect the impact of the mutation on the single-channel current at the free-running yeast membrane potential, the corresponding open probability, or the product of both variables. The latter time-averaged current will be used in short-hand notation as “activity.” Only for a subset of mutants is a classification into more- or less-active channels with respect to the wild-type (WT) reference possible. Hence, yeast complementation assays are suitable for a positive screening of K^+^ channel function, but they bear limited information on the functional properties of the tested channel.

## Materials and methods

### Constructs

The coding sequence of Kcv_PBCV1_ was cloned into the expression vector pEXP5-CT/TOPO using the pEXP-5-CT/OPOTM TA Expression Kit (Thermo Fisher Scientific, Waltham, MA), following the manufacturer’s instructions. All KcvL94X mutants were introduced via site-directed mutagenesis polymerase chain reaction (PCR) (21,22), using Phusion-DNA-Polymerase (Thermo Fisher Scientific) according to the manufacturer’s specifications. PCR products were separated by electrophoresis in a 1.5% agarose gel in 1× Tris-acetate EDTA (TAE) and purified using the Zymoclean Gel DNA Recovery Kit (Zymo Research Europe, Freiburg, Germany) for gel extraction. The extracted DNA was treated with FastDigest DpnI (Thermo Fisher Scientific) according to the manufacturer’s instructions in order to digest the template DNA. Subsequently, 400 ng of DNA was added to 50 *μ*L of chemically competent DH5*α* cells and incubated on ice for 20–30 min. For transformation, a heat shock was performed for 45–60 s at 42°C with a subsequent immediate transfer on ice for 2 min. Thereafter, 250 *μ*L of SOC medium (20 g/L tryptone, 5 g/L yeast extract, 8.5 mM NaCl, 2.5 mM KCl, 100 mM MgCl_2_, and 20 mM glucose (pH 7)) was added and cells were incubated on a shaker for 1 h at 37°C and 200 rpm before being plated on LB + amp agar plates (10 g/L tryptone, 5 g/L yeast extract, 5 g/L NaCl, 50 *μ*g/L ampicillin, and 20 g/L agar (pH 7)) and incubated at 37°C overnight. Three to four colonies were picked the next day to grow in 4 mL of LB + amp medium (10 g/L tryptone, 5 g/L yeast extract, 5 g/L NaCl, and 50 *μ*g/L ampicillin (pH 7)) overnight at 37°C and 200 rpm. Plasmid DNA was finally purified on the next day using the ZR Plasmid MiniprepClassic-Kit (Zymo Research). For validation of a correct coding region, all constructs were sequenced by Microsynth Seqlab (Göttingen, Germany).

### Yeast complementation assay

Yeast complementation was performed as described previously (9). The Kcv_PBCV1_ gene was cloned into the *Eco*RI and *Xho*I site of a modified version of the plasmid pYES2 (Invitrogen, Karlsruhe, Germany) in which the GAL4 promoter was replaced with the Met-25 promoter (23). Different point mutations were inserted via the QuikChange site-directed mutagenesis method (Stratagene, La Jolla, CA), and the resulting constructs were checked by DNA sequencing. Growth rescue experiments were performed as described by Minor et al. (23). The yeast strain SGY1528 (Mat *a ade* 2–1 *can* 1–100 *his* 3–11,15 *leu* 2–3,112 *trp* 1–1 *ura* 3–1 *trk* 1::*HIS3 trk* 2::*TRP1*) (24), which lacks the two endogenous K^+^ transporters Trk1p and Trk2p, was kindly provided by Dr. Minor (UCSF, San Francisco, CA). For the complementation assay, cells were grown in parallel on agar plates or in liquid cultures in synthetic defined arginine phosphate (SDAP) medium (25) composed of 10 mM arginine, 2% glucose, 1 mM MgSO_4_, 0.1 mM CaCl_2_, 1% trace elements (13 *μ*M FeNaEDTA, 8 *μ*M H_3_BO_3_, 0.25 *μ*M CuSO_4_, 0.6 *μ*M KI, 2.7 *μ*M MnSO_4_, 1 *μ*M Na_2_MoO_4_, 2.5 *μ*M ZnSO_4_, 0.5 *μ*M CoCl_2_, and 0.5 *μ*M NiCl_2_), 1% vitamins (2 *μ*g/L biotin, 400 *μ*g/L Ca-pantothenate, 2 *μ*g/L folic acid, 200 *μ*g/L inositol, 400 *μ*g/L niacin, 200 *μ*g/L *p*-aminobenzoic acid, 400 *μ*g/L pyridoxine hydrochloride, 200 *μ*g/L riboflavin, and 400 *μ*g/L thiamine hydrochloride), 50 mg/L histidine, 50 mg/L tryptophan, and 50 mg/L leucine. The required K^+^ concentration of 0.5 mM, 1 mM, and 100 mM was added as KCl; pH was adjusted to 6 with 85% H_3_PO_4_. Cell growth was monitored at 30°C for 3 days.

For growth in liquid cultures the aforementioned selection medium with a low 0.5 mM K^+^ concentration was used, and the optical density was measured at 600 nm (OD_600_). Therefore, 0.5 mM K^+^ medium was inoculated with a yeast suspension (prepared and washed in the same manner as for the plates (23)) to a final OD_600_ of 0.1 and incubated directly in sterilized 2-mL cuvettes sealed with laboratory film at 30°C and 230 rpm. After defined times, the OD_600_ was measured using a spectrophotometer. While the OD_600_ value by itself does not discriminate between live and dead cells, the increase of this value over time is an indirect but robust measure of growth.

### Functional reconstitution

For measurements of single-channel activity in planar lipid bilayers the Kcv_PBCV1_ and its L94X mutants were expressed in vitro with a MembraneMax HN Protein Expression Kit (Thermo Fisher Scientific) or Expressway Mini Cell-Free Expression System (Thermo Fisher Scientific) according to the manufacturer’s instructions, with the difference of halving the total volume of the reaction mixture. To enable proper folding of membrane proteins and to avoid protein aggregation, the expression was performed in the presence of nanodisks. These were either already included in the kit (MembraneMax HN Protein Expression Kit), containing dimyristoylphosphatidylcholine (DMPC) lipids, or commercially acquired MSP1D1 nanodisks from Cube Biotech (Monheim, Germany), adjusted to a concentration of 30 *μ*mol for the reaction mixture. The latter have a scaffold diameter of 9–10 nm and are available with different assembled lipids (DMPC, DMPG (1,2-dimyristoyl-*sn*-glycero-3-phosphoglycerol), or POPC (1-palmitoyl-2-oleoylphosphatidylcholine)). A big advantage of nanodisks is the possibility to isolate membrane proteins without contaminations from the bacterial lysate with the help of a histidine tag (His-tag) attached to the membrane scaffold protein (MSP) of the nanodisk. This His-tag allows purification of the nanodisk/channel complex via metal chelate affinity chromatography, using a 0.2-mL HisPur Ni-NTA spin column (Thermo Fisher Scientific). This method allows not only contamination-free expression but is also much faster than expression systems used to produce channel proteins for most bilayer experiments, such as expression in heterologous systems (26). Differing from the manufacturer’s instructions, wash and elution buffer only contained 25 mM and 250 mM imidazole, dissolved in ddH_2_O. Keeping the protein-containing nanodisks in solutions containing salts has been shown to reduce channel activity dramatically (27). For single-channel measurements the first elution fraction was diluted in 250 mM pure imidazole 1:10^2^–1:10^4^, depending on protein activity.

Functional reconstitution of channel proteins in planar lipid bilayers was performed as described previously (28). Bilayers were formed from 1,2-diphytanoyl-*sn*-glycero-3-phosphocholine (DPhPC; Avanti Polar Lipids, Alabaster, AL) by the pseudo-painting/air-bubble technique (29). For single-channel recordings 1–3 *μ*L of the diluted elution fraction was added directly below the bilayer in the *trans* compartment with a bent Hamilton syringe. *Cis* and *trans* chambers were filled with standard recording buffer (100 mM KCl and 10 mM HEPES in ddH_2_O, adjusted to pH 7.0 with KOH). Insertion and orientation of a single-channel protein was monitored during short voltage steps. The pronounced asymmetry of Kcv_PBCV1_ channel conductance allowed determination of channel orientation in the bilayer. The *trans* compartment was grounded, and membrane voltages were applied to the *cis* compartment. When the Kcv_PBCV1_ protein was added to the bilayer from the *trans* side, it exclusively inserted into the membrane with the cytoplasmic side first (26). This means that negative currents in the bilayer recordings correspond to inward currents (= potassium influx) through the channel in the plasma membrane of yeast cells.

Steady-state single-channel currents were recorded over a voltage window from +160 mV to −160 mV in steps of 20 mV for 1–5 min. The latter extended recording times were routinely used for channel mutants with a low open probability to guarantee sufficient opening events for analysis. Both chambers were connected via Ag/AgCl electrodes to the head stage of an L/M-EPC-7 amplifier (List-Medical, Darmstadt, Germany). Currents were filtered with a 1-kHz 4-pole Bessel filter before digitization with a sampling frequency of 5 kHz (LIH 1600; HEKA Elektronik, Lambrecht, Germany).

### Single-channel analysis

Single-channel traces were analyzed as in previous studies (30,31) with the custom-made analysis tool KielPatch version 3.2 (ZBM/2011), freely available at the Center for Biochemistry and Molecular Biology, CAU Kiel, Germany (http://www.zbm.uni-kiel.de/aghansen/software.html). The unitary current amplitudes (*i*) were measured directly from the recorded traces as a difference between baseline and open-channel level. The unitary conductance, *g*, was obtained from single-channel *i/V* relations by a linear fit through data between 0 mV and +100 mV. To determine the open probabilities, *P*_O_, an automated higher-order Hinkley jump detector (HOHD) in the software (30,31), which confidentially detects opening and closing transitions of a channel, was employed. This detector allows the idealization of single-channel current fluctuations and, thus, the determination of open and closed dwell times. The open probability was then calculated by dividing the sum of all open dwell times (*t*_open_) by the total time of recording (*t*_total_). A detailed description of the HOHD and the time resolution of the event detection is given in (30).

### Statistics and correlation analysis

Statistical comparison of mutated channels and Kcv WT channels were performed with the two-tailed unpaired Student’s *t*-test, using Microsoft Excel. The respective numbers of measurements (*n*) are provided in figure legends and tables. *p* values of <0.05 were considered statistically significant. Calculation of Pearson’s correlations and corresponding plots were created using Wolfram Mathematica 13.3.1.0 (Wolfram Research, Champaign, IL, 1988–2023).

## Results

**Figure 2:**
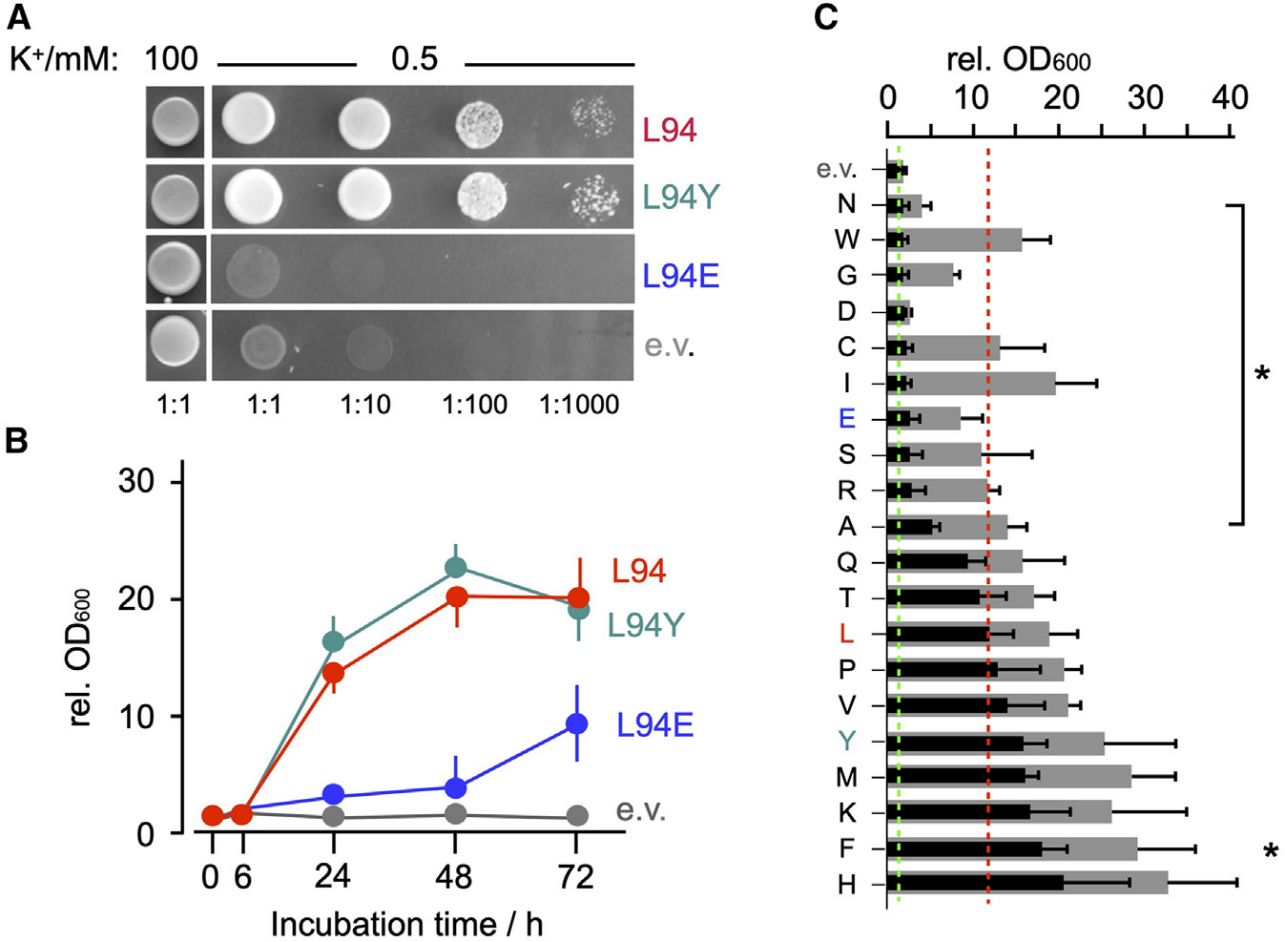
Complementation assays of yeast strain SGY1528 transfected with pYES2, Kcv_PBCV1_, and Kcv_PBCV1_ L94X. (*A*) Drop test showing potassium requirement of trk1Δtrk2Δ double-knockout yeast strain SGY1528 for growth in 100 mM K^+^ and rescue of growth in low-K^+^ medium with 0.5 mM K^+^ by transfection of cells with WT Kcv_PBCV1_ channel (L94) and selected mutants with high (L94Y) and low (L94E) complementation efficiency. Cells transfected with empty vector (e.v.) served as negative control. Cells were spotted as serial dilutions from an OD_600_ = 1 on agar plates at 3 *μ*L per spot. (*B*) Growth assay in liquid culture of SGY1528 cells transfected with empty vector as well as WT channel (L94) and mutants with high (L94Y) and low (L94E) complementation efficiency in 0.5 mM K^+^ selective medium. OD_600_ were measured at time of inoculation (*t* = 0) as well as 6, 24, 48, and 72 h later. Data report mean fold changes in OD_600_ over respective value at time of inoculation (mean ± SD of *n* = 3 experiments). (*C*) Yeast growth assays in liquid culture as in (*B*) of SGY1528 cells transfected with Kcv_PBCV1_ and its L94X mutants, where capital letters on the left represent the amino acid of the corresponding mutation abbreviated with the one-letter code. Yeast growth in liquid medium as in (*B*) with 0.5 mM KCl is shown as bars measured by the corresponding OD_600_ after 24 h (*black bars*) and 48 h (*gray bars*); the corresponding values are listed in Table S1. The OD_600_ level monitored with control cells (empty vector, e.V.) and with the WT channel (L94) after 24 h of incubation is indicated by red and green dashed lines, respectively. Data are mean values ± SD of three independent measurements. Mutants that over 24 h of incubation generate a significantly (*p* < 0.05) reduced or elevated growth relative to the WT channel are indicated by an asterisk.

Motivated by data from previous yeast complementation assays, which indicated an importance of the last amino acid L94 in the viral channel Kcv_PBCV1_ for channel function (9), we mutated the respective leucine into all remaining 19 proteinogenic amino acids. All mutant channels were functionally expressed in the SGY1528 yeast strain (8), which lacks the two endogenous K^+^ uptake systems Trk1p and Trk2p. The ability of the WT channel and its L94X mutants to rescue growth of the SGY1528 yeast strain was examined on agar plates and in liquid culture with a low K^+^ concentration of 0.5 mM. Fig. 2, *A* and *B* show representative drop tests and the corresponding OD_600_ measurements in liquid culture from yeast cells expressing the WT Kcv_PBCV1_ channel (L94) or two mutants, L94E and L94Y. As expected from previous experiments (9), the yeast mutants transfected with an empty vector are unable to grow in this selective low-K^+^ medium. After transfection with the functional Kcv_PBCV1_ channel, growth in low-K^+^ medium on agar plates as well as in liquid medium is restored. Fig. 2 *B* shows that growth of cells expressing the WT channel becomes evident >6 h after incubation and approaches saturation at 48 h. The high density of yeast colonies on agar and the strong increase in the OD_600_ value imply that the L94Y mutant also rescues growth presumably even better than the WT channel. Cells expressing the L94E mutant, on the other hand, show over 48 h hardly any growth on the agar plates as well as in liquid medium beyond the performance of the negative controls transfected with the empty vector only.

Experiments performed as in Fig. 2 *B* were repeated with WT Kcv_PBCV1_ as well as with all possible mutants, in which L94 was replaced by one of the remaining 19 proteinogenic amino acids. The OD_600_ values measured after 24 and 48 h of incubation in selective low-K^+^ medium show a large variety of growth phenotypes. A few mutants (Q, T, and P) show a compatible efficiency to the WT channel in rescuing yeast growth. Other mutants such as Y, M, K, F, and H perform presumably better over 24 h of incubation, i.e., a time at which growth of cells with the WT channel is not yet saturated (Fig. 2 *B*). Many mutants, on the other hand, show significantly reduced or delayed rescue efficiency. Worth mentioning are two mutants, L94N and L94D. In cells expressing these mutants, growth remained low even over 48 h of incubation; the respective OD_600_ values barely exceeded the value obtained for cells transfected with the empty vector only. Based on the assumption that the rescue of yeast growth depends on the function of the Kcv channel, we assume that the latter might not be functional.

### Effect of L94X mutations on single-channel properties

**Figure 3:**
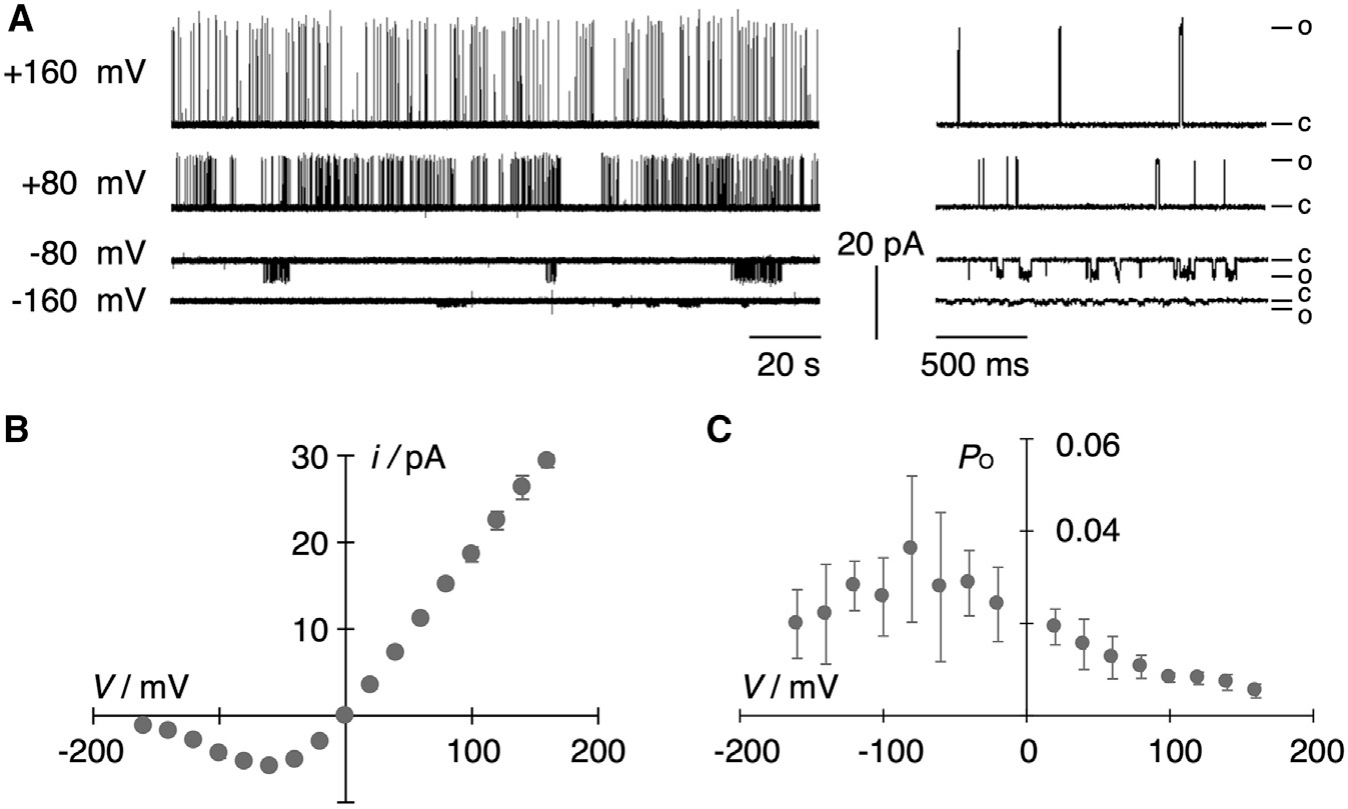
Single-channel properties of Kcv_PBCV1_. (*A*) Representative single-channel traces of Kcv_PBCV1_ from +160 mV to −160 mV in 80-mV steps with two different temporal resolutions. Traces on an extended time axis (*right side*) show individual gating events in detail and do not reflect the general open probability. Letters c and o mark the closed and open level, respectively. For better visualization, data were subsequently filtered with 500 Hz. (*B*) Open-channel *i*/*V* curve for Kcv_PBCV1_. (*C*) Open probability curve for Kcv_PBCV1_. Data are mean values ± SD of eight independent measurements (*n* = 8). Measurements were performed under symmetrical conditions with 100 mM KCl and 10 mM HEPES at pH 7.

To correlate the different degree of yeast growth rescue with functional properties of WT Kcv_PBCV1_ and its mutants, they were all synthesized in vitro into nanodisks and reconstituted in planar lipid bilayers (26). Fig. 3 *A* shows for reference typical fluctuations of the WT channel in a DPhPC bilayer in symmetrical 100 mM KCl buffer where it exhibits its characteristic single-channel features (32). This includes well-resolved channel fluctuations at voltages positive of −50 mV giving rise to a near linear unitary conductance of 174 ± 9 pS (*n* = 37). With progressive hyperpolarization, the channel open state is increasingly interrupted by short closures, resulting in a flicker-type gating (Fig. 3 *A*) together with a gradual reduction of the open-channel current (Fig. 3, *A* and *B*). This phenomenon of decreasing unitary current amplitudes with increasing hyperpolarizing voltages has been attributed to a rapid voltage-dependent gating (33,34). Because of this flicker-type gating, the full (or “true”) open-channel amplitude of the channel (*i*_true_) is no longer resolved; the recording instrumentation only provides the “measured” open-channel amplitude (*i*_meas_) (34). Since the same phenomenon of flicker gating with an asymmetry of the Kcv_PBCV1_ conductance is also observed in measurements of this channel in mammalian cells, we can interpret negative currents from bilayer recordings as an inward current in the *in vivo* situation (35).

Analysis of WT channel activity as a function of voltage shows that Kcv_PBCV1_ exhibits an overall low open probability (Fig. 3, *A* and *C*). Typically, the *P*_O_ value increases about 10-fold from a very low value of 0.005 ± 0.002 at +160 mV in a weakly voltage-dependent manner to a value of 0.034 ± 0.012 at −160 mV (Fig. 3 *C*).

In the next step, all mutants from Fig. 2 *C* were reconstituted into planar lipid bilayers (26); as a negative control the mutant K29P, which is non-functional in complementation assays (36), was used. While reconstitution of the K29P mutant resulted in eight independent attempts in no measurable channel activity, we found that replacing L94 by 19 other amino acids generated functional channels in all cases.

**Figure 4:**
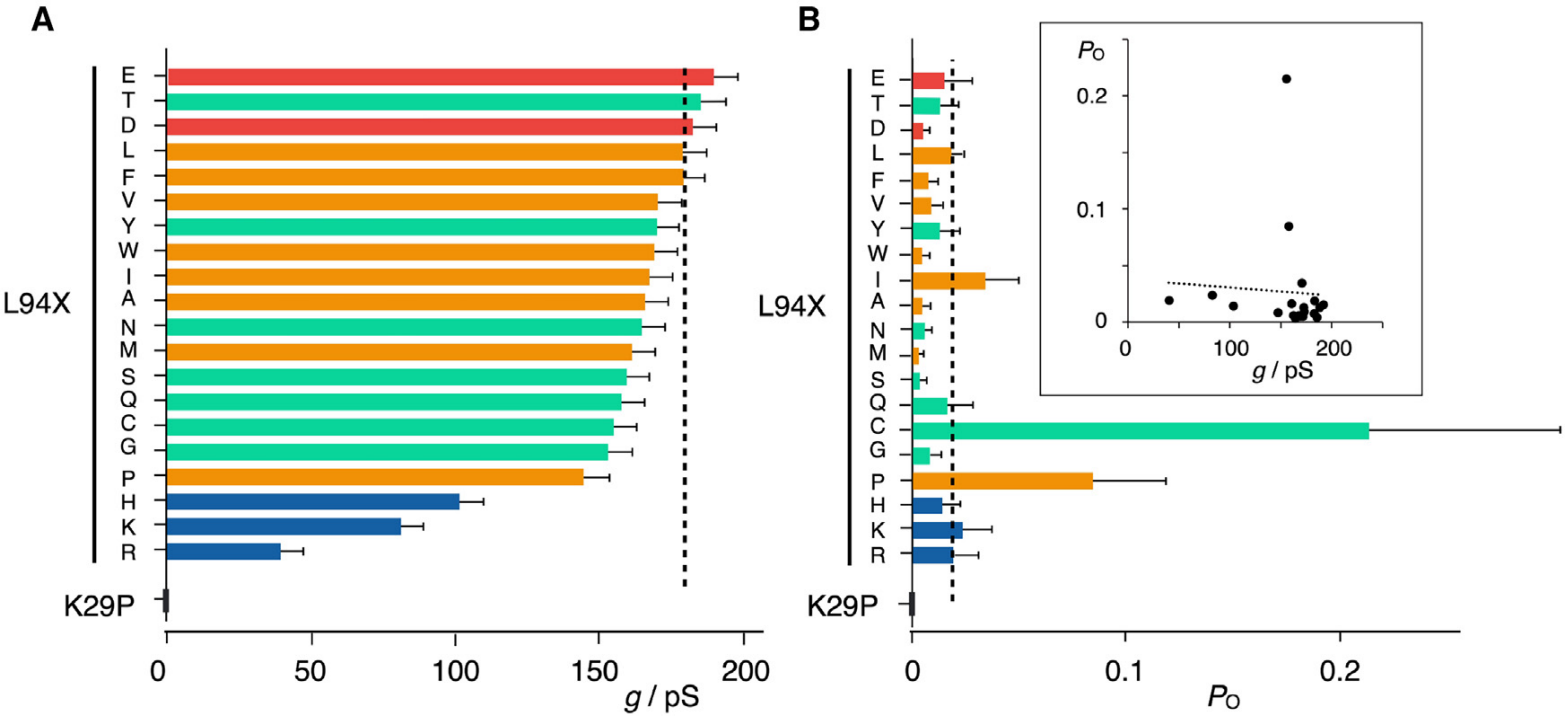
Unitary single-channel conductance of fully open channels and open probability of Kcv_PBCV1_ and its mutants. (*A*) Unitary conductance, *g*, of Kcv_PBCV1_ channel (sorted from high to low) as a function of the amino acid in position 94, given in one-letter code on the left, and of mutant K29P. The respective conductance was obtained from a linear fit to the *i*/*V* data between 0 mV and +160 mV. (*B*) The corresponding mean open probability for Kcv_PBCV1_ and mutants as in (*A*). Inset: scatterplot of *P*_O_ and conductance for WT channel and its mutants; Pearson’s correlation for both parameters is shown as a dashed line. The mean *P*_O_ value was calculated from the open probability monitored over the entire voltage window between −160 and +160 mV. Different colors in (*A*) and (*B*) represent four different amino acid properties: green, polar/neutral; yellow, non-polar/hydrophobic; blue, basic; red, acidic amino acids. The level of unitary conductance and *P*_O_ of wild-type (L) Kcv_PBCV1_ is marked with dashed lines. Data in (*A*) and (*B*) are mean values ± SD of *n* independent measurements listed in Table S1.

For an initial overview on the impact of mutations on channel function, we analyzed the conductance at positive voltages. In the case of Kcv channels, this is a good measure of the fully open channel and in general a fingerprint of ion-channel function. We also estimated the global open probability of the mutants between ±160 mV, ignoring the moderate voltage dependency of this parameter (e.g., Fig. 3 *C*). The two functional parameters for the WT channel and its L94X mutants are plotted in Fig. 4 *A* and *B*, respectively and listed in Table S1.

The data in Fig. 4 *A* and *B* provide two important kinds of information: First, all mutants including L94N and L94D, which reveal no significant rescue of yeast growth, generate a measurable channel activity. Hence, a failure of K^+^ channel mutant in the yeast rescue assay is not per se an indication for a non-functional channel. Second, the properties of the amino acid side chains at the entry to the cavity have, as expected (9), a specific impact on channel function. Depending on the amino acid, this affects the unitary conductance and/or open probability (Fig. 4, *A* and *B*). A comparison of *A* and *B* in Fig. 4 indicates that the impacts of mutations on channel conductance and open probability are not directly coupled (Fig. 4 *B*, *inset*); the Pearson correlation coefficient between the two data sets is, at −0.05, close to zero.

### Causal relationship between single-channel properties and yeast growth

Next, we wanted to understand whether the primary parameters of functional single-channel current, open probability, and/or the product of both are sufficient to explain the observed yeast rescue response.

It is established that K^+^ uptake into yeast is driven by a H^+^ ATPase, which hyperpolarizes the membrane potential and generates in this manner a passive influx of K^+^ into the cells (1). Because negative currents from bilayer recordings are a measure of the inward current in the *in vivo* situation (35), we can take the open probability as well as the single-channel current of Kcv_PBCV1_ and its mutants at negative voltages as an approximation for the physiological impact of the channel on yeast growth. For this analysis, we chose the single-channel current and its open probability at −140 mV. This voltage is in the range of the free-running membrane potential of yeast cells in medium with low K^+^ (37,38) and, hence, relevant for the uptake of K^+^ for yeast growth. The respective open probability at −140 mV (*P*_O,−140_) for WT channel and mutants (listed in Table S1) can be directly taken from *P*_O_/*V* relationships as shown in Fig. 3. Assuming that also in the physiological context fast gating will serve as a low-pass filter for the K^+^ flux into the cell, we use the measured current amplitude *i*_meas,−140_ directly from the *i*/*V* relations at −140 mV (Table S1). Because the unitary current *i*_meas,−140_ of the channel is due to fast gating not reaching full opening in this voltage window (33,34), we use an alternative value for estimating the physiologically relevant current. Because *i*_meas,−140_ is very small and, due to its flicker gating, difficult to determine precisely, we additionally estimated the fully open channel current at this voltage (*i*_true,−140_). Previous work had shown that this value can be approximated in symmetrical 100 mM KCl buffer from a linear extrapolation of the *i*/*V* relation at positive voltages (39); the values for the WT channel and its mutants are listed in Table S1.

**Figure 5:**
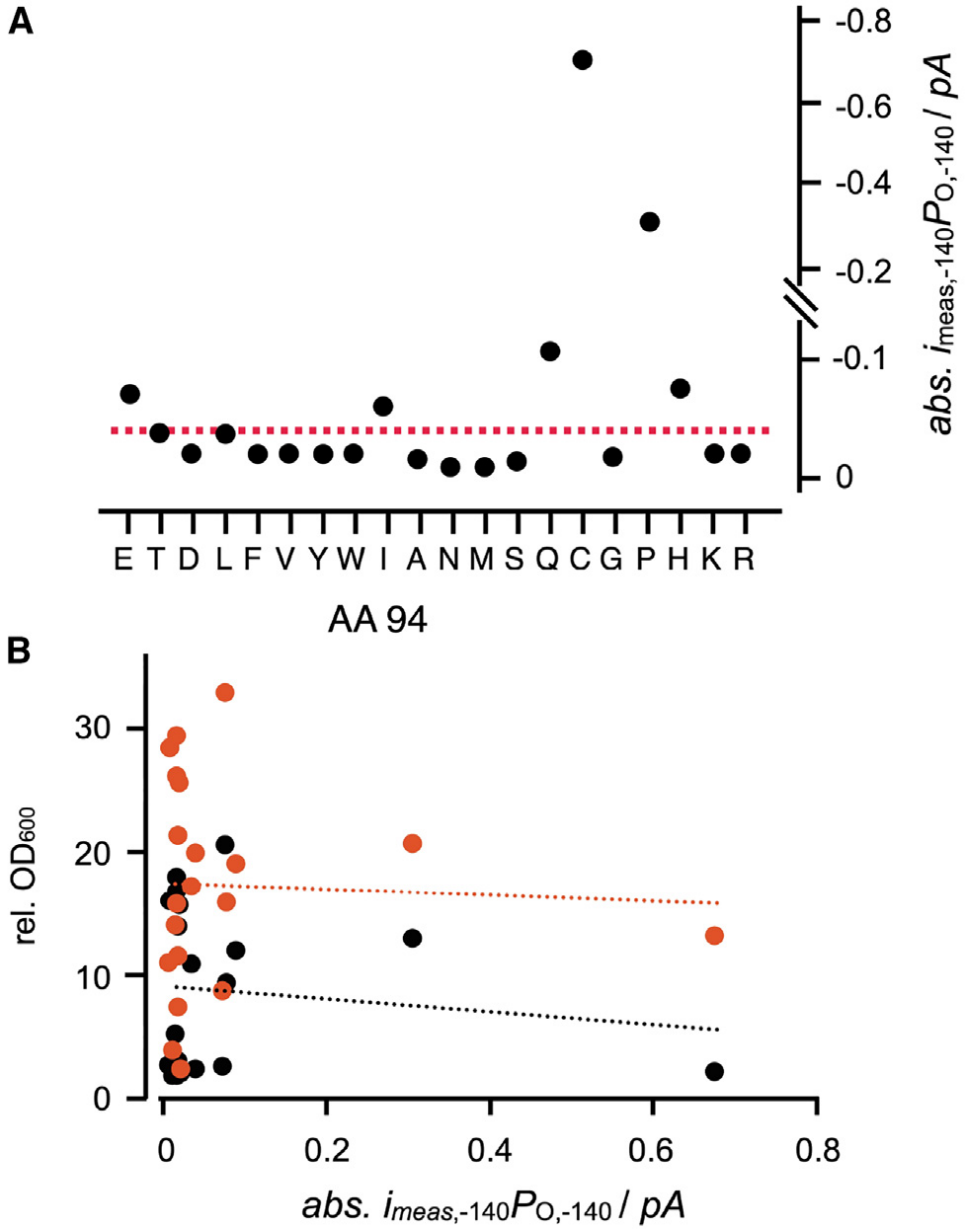
Time-averaged current at physiological membrane potential of L94X mutants in relation to potency of the mutants to stimulate yeast growth. (*A*) Time-averaged absolute current (*i*_meas,−140_ × *P*_O,−140_) of WT channel and L94X mutants calculated from measured unitary channel current (*i*_meas,−140_) and corresponding open probability (*P*_O,−140_) at −140 mV. The values *i*_meas,−140_ and *P*_O,−140_ were directly obtained from their single-channel *i*/*V* and *P*_O_/*V* relations as in Fig. 3. (*B*) The fold changes in the OD_600_ value over 24 h (*black*) and 48 h (*orange*) of growth in liquid medium with 0.5 mM K^+^ (data from Fig. 2) for the WT and the L94X mutants are plotted as a function of the absolute time-averaged currents in (*A*). The Pearson correlation coefficients for the channel properties and the growth data (*dashed lines*) are small for both 24 h (−0.12) and 48 h (−0.05) of growth (Table S2).

Following this rationale, we plotted in Fig. 5 *A* the time-averaged current (= activity), i.e., the product of the measured single-channel amplitude (*i*_meas_) and the open probability (*P*_O_) of the WT and the mutant channels at −140 mV (*i*_meas,−140_ × *P*_O,−140_). Scrutiny of the *i*/*V* and *P*_O_ relations from WT and L94X mutant channels (e.g., Fig. 3) shows that the values for *i*_meas_ and *P*_O_ approach saturation at this voltage. Hence, even if the real free-running membrane voltage of yeast cells is presumably more negative than −140 mV (1), the functional parameters of the channels are, at the chosen value, also a good approximation of their activity at even more negative voltages. The data in Fig. 5 *A* indicate that some mutants (e.g., L94A, M, N, and S) should cause a lower growth rate than the WT channel while others, such as L94Q and even more so L94P and C, should greatly augment growth.

To test the quantitative relationship between channel activity and yeast growth, the latter value was plotted for the L94X mutants as a function of the corresponding time-averaged currents at −140 mV (*i*_meas,−140_ × *P*_O,−140_) (Fig. 5 *B*). Inspection of the data, however, shows no meaningful correlation between channel function and cell growth in low-K^+^ buffer over 24 or 48 h in liquid medium (Table S2). The same analysis repeated by considering the true single-channel current at −140 mV (*i*_true,−140_) also showed low correlation between yeast growth and the time-averaged true current (*i*_true,−140_ × *P*_O,−140_) (Table S2).

Finally, we also tested the remote possibility that the channel protein might have an inverse orientation in the plasma membrane of yeast cells. To mimic this condition, we analyzed the degree of growth rescue as a function of *i*_true,+140_ × *P*_O,+140_. This analysis also reveals no appreciable correlation between the electrical data and the growth rescue results (Table S2).

From these analyses, we must conclude that the degree of yeast rescue in a growth assay by Kcv_PBCV1_ and its L94X mutants cannot quantitatively be derived from the most significant functional parameters of the channels alone, namely the single-channel current and/or open probability, or their product, from single-channel recordings.

**Figure 6:**
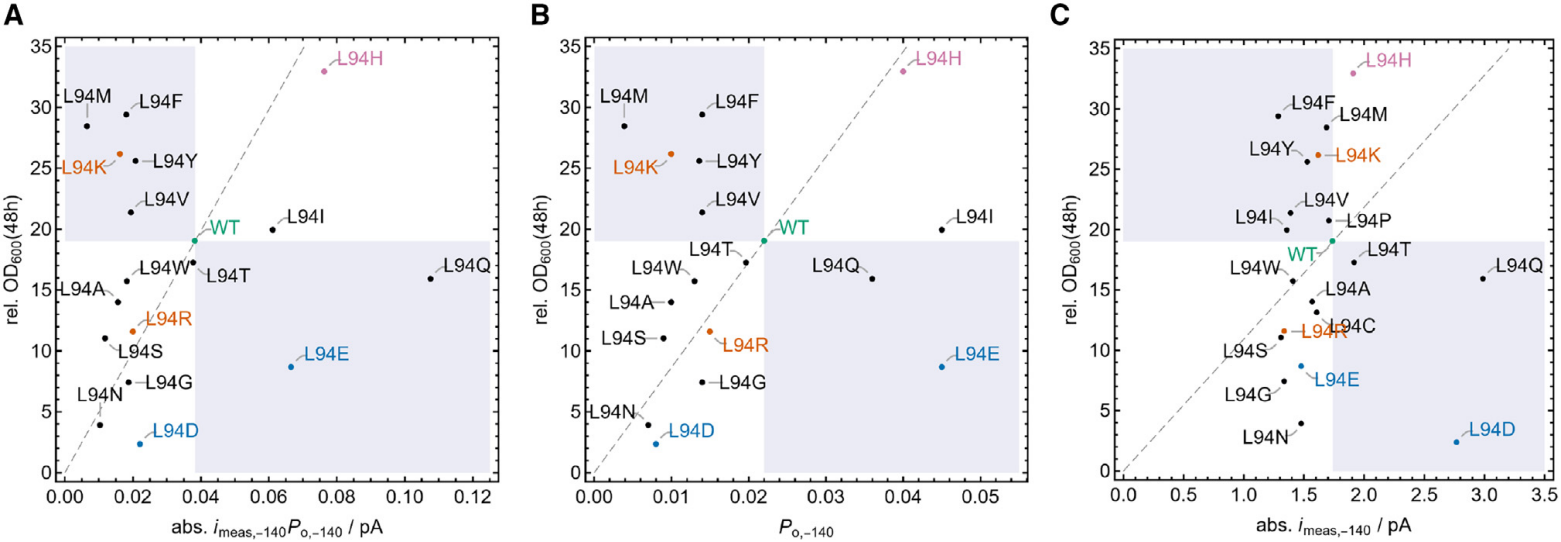
Relative OD_600_ after 48 h against absolute time-averaged current (abs. *i*_meas,−140_ × *P*_O,−140_) (*A*), open probability (*P*_O,−140_) at −140 mV (*B*), and absolute measured unitary channel current (abs. *i*_meas,−140_) (*C*) for WT and all L94X mutants. Currents are expressed as absolute numbers (abs. *i*_*meas*,−140_) to make comparison between different panels easier. White areas show a positive classification, and gray areas indicate a negative classification. Cationic mutants are indicated in orange, anionic mutants in blue, histidine in pink, and WT in green. The full data set including all mutants is presented in Figs. S1–S3, and statistical correlation data are summarized in Table S3.

Even in the absence of a quantitative correlation, it is possible to inspect the data in the sense of a classification. We therefore plotted in Fig. 6 OD_600_ (48 h) vs. |*i*_meas,−140_| (absolute value taken for clarity), *P*_O,−140_, and their product in a way to allow for distinguishing between channels that are more or less active than the WT (right and left region separated by a vertical line dissecting the WT point, respectively). If the OD_600_ corresponds to this trend (higher OD_600_ for more-active mutants than the WT, lower OD_600_ for less-active ones, measured by regions above or below a horizontal line dissecting the WT point, respectively), points are found in the white areas in Fig. 6. Residues violating this classification are in the gray-shaded areas. Fig. 6 now shows this classification for the product (Fig. 6 *A*) as well as for separate open probabilities (Fig. 6 *B*) and apparent currents (Fig. 6 *C*). The representation has been truncated here at some upper activity limit for clarity; the full plots including the missing mutants L94P and L94C are presented in Fig. S1. All plots including standard errors are also shown in Fig. S2, indicating that the classification is indeed possible within the apparent errors.

In this classification sense, the picture is not as unspecific as that obtained from direct quantitative correlation. From the product representation (Figs. 6 *A*, S1 *A*, and S2), we find that L94 mutants A, W, T, R, S, G, N, D, I, H, and P are correctly classified, whereas M, F, K, Y, V, E, Q, and C are not. Remarkably, there is no consistent classification of both positively (K and R) and negatively (D and E) charged residues. Both are found in different areas, either positively or negatively classified. It is also interesting to note that the classification with the open probability only (Figs. 6 *B* and S1 *B*) yields very similar results compared to the product, whereas the apparent currents (Figs. 6 *C* and S1 *C*) are much worse related to the yeast growth. The same results were obtained when the analysis was performed with the estimated true current at −140 mV instead of the measured *i*_meas_ value (Fig. S3).

According to this analysis, more than 50% of the mutant channels meet the expectations in that growth of the yeast mutants is related to the activity of the channel; the degree of yeast growth is in these cases a function of the influx of K^+^, which passes through the Kcv channel into the cells. Under physiological conditions, the activity of a channel is determined by the product of its single-channel amplitude and the open probability at the prevailing membrane potential. Analysis of Fig. 6, where the impact of the mutants on yeast growth was calculated separately for the open probability *P*_O,−140_ (Fig. 6 *B*), the single-channel current *i*_meas,−140_ (Fig. 6 *C*), and the product of both parameters (Fig. 6 *A*), suggests that the open probability is in this context the prevailing factor. Replotting the data from Fig. 6 *A* and *B* with the standard errors for the functional parameters of the channel and for yeast growth in Fig. S2 supports this scenario. The definition of the functional properties *i*_meas,−140_ × *P*_O,−140_ and *P*_O_ of the mutants that are positively correlated with yeast growth in the classification sense are significantly separated from those that do not show this effect (Fig. S2).

**Figure 7:**
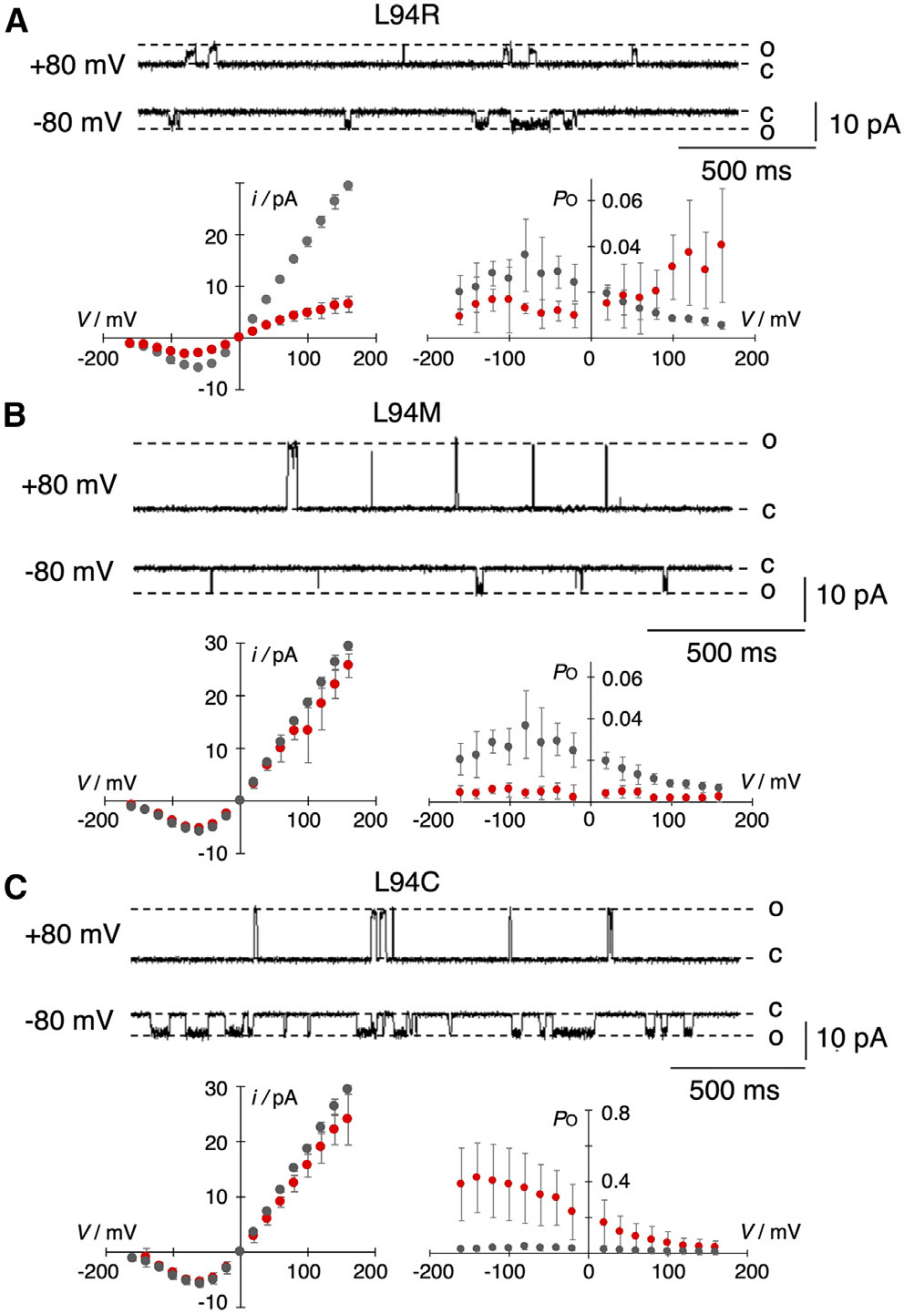
Single-channel properties of three Kcv_PBC1_ L94X mutants. Representative single-channel traces of Kcv_PBCV1_ L94R (*A*, *top row*), L94M (*B*, *top row*), and L94C (*C*, *top row*) at ± 80 mV. C and O mark the closed and open levels, respectively. The plots below current traces show the corresponding *i*/*V* (*left*) and *P*_O_/*V* (*right*) relationship. The data of the WT channel are included in each plot as a reference in gray. The respective data from the mutant are shown in red. Data are mean values ± SD of *n* independent measurements (Kcv_PBCV1_, *n* = 8; L94R, L94M, and L94C, *n* = 3 each). Measurements were performed under symmetrical conditions with 100 mM KCl and 10 mM HEPES at pH 7.

Collectively, the data suggest that yeast growth is in this experimental setting more affected by the open probability and to a lesser extent by the single-channel currents at the physiological membrane voltage. This finding is not surprising because fast gating at negative voltages basically abolishes differences in the unitary conductance between WT and mutants. This is apparent in the *i*/*V* relation of the L94R mutant in Fig. 7. However, even a multilinear model using currents, open probability, and product of both as independent variables for fitting yeast OD_600_ data does not yield a quantitatively applicable relationship (*R*^2^ of 0.106 for *i*_meas_ and 0.070 for *i*_true_), further emphasizing that other hidden yeast-specific variables affect the observed response.

### Effect of different amino acids on unitary current and open probability

The systematic exchange of L94 with all possible amino acids shows that their flavor at the entry to the cavity has, depending on the amino acid, an impact on the unitary conductance and/or open probability (Fig. 4, *A* and *B*). Fig. 7 *A*–*C* illustrates in more detail three examples in which a mutation decreases the unitary conductance (L94R), decreases the open probability (L94M), or increases the open probability (L94C).

**Figure 8:**
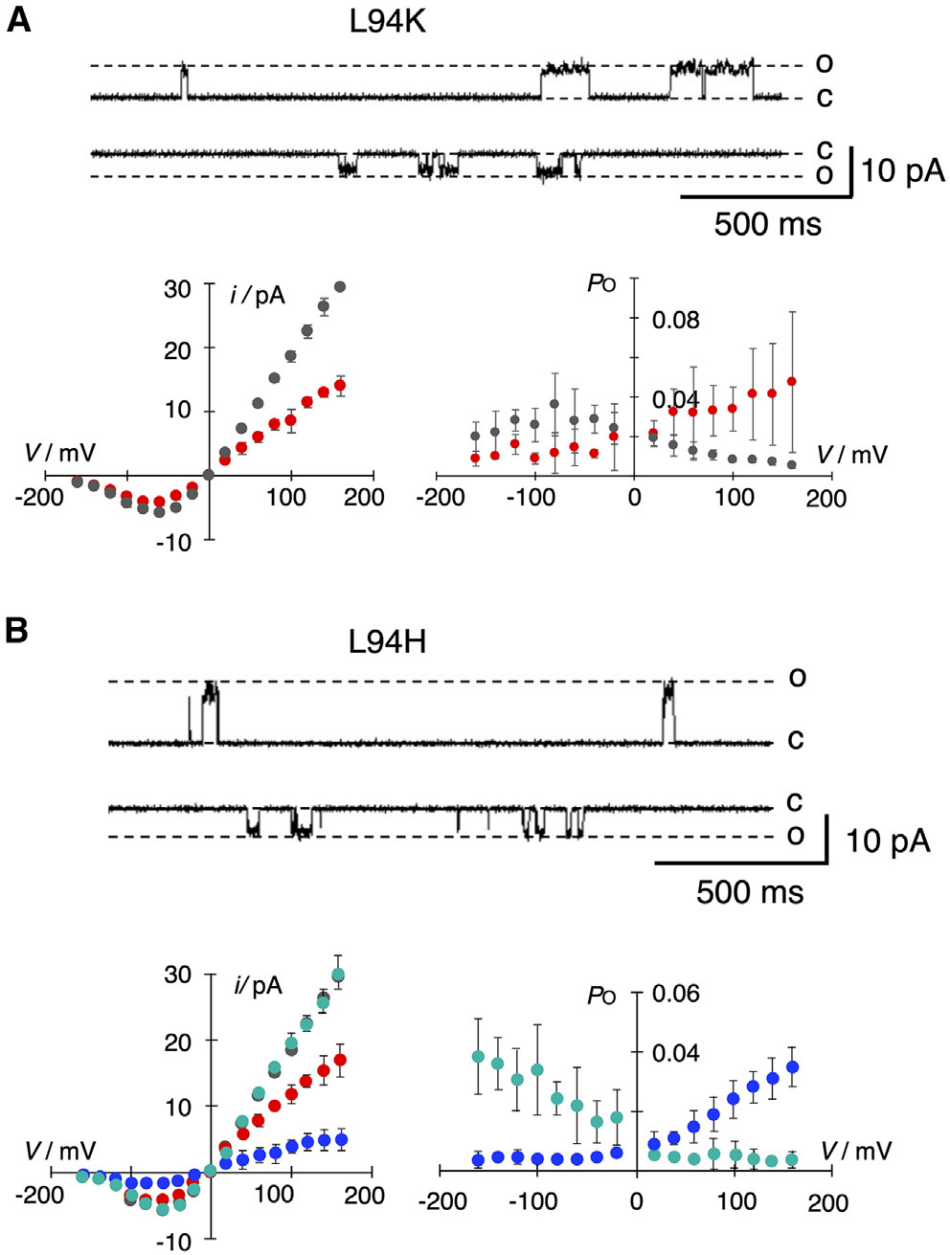
Impact of charge in position 94 for channel function. Representative single-channel traces of Kcv_PBCV1_ L94K (*A*, *top row*) and L94H (*B*, *top row*) at ±80 mV. C and O mark the closed and open levels, respectively. Plots below current traces show the corresponding *i*/*V* (*left*) and *P*_O_/*V* (*right*) relationships. Data of the WT channel are included in each plot as reference in gray. Data from mutants are shown in red for pH 7 and blue and green for pH 4 and pH 9, respectively. Data are mean values ± SD of *n* independent measurements (Kcv_PBCV1_, *n* = 8; L94K, *n* = 3; L94H, *n* = 4 (pH 7), *n* = 4 (pH 4), and *n* = 4 (pH 9)). Measurements were performed under symmetrical conditions with 100 mM KCl and 10 mM buffer (pH 7: HEPES; pH 9: TAPS; pH 4: 90 mM KCl; pH 4: 10 mM CH_3_COOK).

Channel fluctuations of L94R at ±80 mV and the corresponding mean *i*/*V* curve show that the cationic amino acid causes a 4.3-fold decrease in the unitary conductance (Fig. 7 *A*). A reduced unitary conductance was also obtained when L94 was replaced by arginine (R) or histidine (H), suggesting that a positive charge in this position lowers the unitary conductance (Fig. 4 *A*). To test this assumption, the L94H mutant was measured in a buffer at either pH 4 or pH 9, which fully protonates or deprotonates the imidazole side chain of histidine, respectively. In agreement with the assumed negative effect of a positive charge, the unitary conductance of the L94H mutant decreases at pH 4 and increases to that of the WT channel at pH 9 (Fig. 8). The impact of charge at this site on the unitary conductance is further supported by the finding that anionic amino acids slightly elevate the conductance with respect to the WT channel (Fig. 4 *A*). The conductance of L94E is indeed significantly (*p* < 0.002) higher than that of the WT channel.

The L94R mutant also has a small but interesting impact on the voltage dependency of channel activity (Fig. 7 *A*). While the WT channel is weakly inward rectifying, the voltage dependency of the L94R mutant is inverted (Fig. 7 *A*). This effect is related to the positive charge in this position because the same inversion in voltage dependency is found in the L94K mutant (Fig. 8 *A*). Also, in agreement with this hypothesis, the voltage dependency of *P*_O_ is inverted in the L94H mutant from an inward rectification at pH 9 to an outward rectification at pH 4 (Fig. 8 *B*).

The replacement of L94 by methionine (L94M) has, like many other mutations, no or only a small impact on the unitary conductance (Fig. 7 *B*) but further reduces the already low open probability of the channel. The highest mean *P*_O_ value measured in the mutant at −100 mV is, at 0.005 ± 0.003, six times lower than the corresponding value in the WT channel. The *P*_O_/*V* relationship of the L94M mutant suggests that the mutation abolishes the weak voltage dependency of the channel and reduces *P*_O_ over the entire voltage window to the small value of the WT channel at positive voltages.

An inverse behavior is seen with the L94C mutant, in which the voltage-dependent opening of the channel at negative voltages is greatly augmented. While the unitary conductance is not strongly affected, the maximal *P*_O_ value at negative voltages is, at 0.4 ± 0.2, ca. 10-fold higher than that in the WT channel (Fig. 7 *C*). The same stimulating effect of the L94C mutation was recorded before and after adding 5 mM dithiothreitol (DTT) to the buffer on the *cis* and *trans* side of the channel (data not shown). The insensitivity to DTT suggests that a formation of sulfhydryl bonds can be ruled out as a responsible mechanism for the increase in open probability.

## Discussion

We have replaced one critical amino acid in the small Kcv_PBCV1_ channel by all other proteinogenic amino acids and have examined its functional impact by two complementary methods, namely single-channel recordings and yeast complementation assays. The first message from these experiments is that unitary current as well as the open probability are distinctly sensitive to the physicochemical flavor of the amino acid at the cytosolic entrance to the cavity. Surprisingly, these altered functions can only be explained to a limited extent by the chemical nature of the individual amino acids at this site. Only the unitary conductance and the direction of voltage dependency of the channel can be coherently explained by the charge of the respective amino acid side chain. The functional impact of other amino acid substitutions, which for example strongly elevate (L94C and L94P) or reduce (e.g., L94M, L94D, and L94S) the open probability, cannot be explained by such a single-variable relationship. Neither the charge nor the hydrophobicity nor the size of the amino acid in position 94 alone seems responsible for the impact on the open probability. Hence, even amino acid substitutions that are considered conservative differ significantly in their impacts on channel function. This underpins that the functional impact of an amino acid on channel function cannot be reduced to a single chemical property. This is presumably not only the case for the Kcv channel but for the analysis of structure-function correlates in any protein.

Because growth of the trk1Δtrk2Δ double-knockout yeast strain in low-K^+^ medium requires the proper function of a heterologous K^+^ channel (6), we anticipated that the functional properties of Kcv channel mutants should be reflected in their degree of growth rescue. The systematic analysis of 20 channel variants shows that this simple assumption does not hold true as a generally applicable quantitative correlation. Direct comparison of the basic single-channel properties of a mutant and its potency to restore yeast growth exhibits no unequivocal pattern for a relationship between these values. For example, there is no apparent indication that a growth promotion by the L94F, L94M, L94Y, and L94H mutants is related to a high unitary conductance and/or open probability. Hence, we must conclude that other hidden variables contribute to the performance of yeast growth rescue.

At this point, it should be mentioned that the plasma membrane of yeast cells is much more complex than the simple phosphatidylcholine lipid membrane used in the artificial lipid bilayer system. Typical for eukaryotes, the cell membrane of yeast is made of diverse phospholipids with various physicochemical flavors, which are mostly asymmetrically distributed between the inner and outer membrane leaflet (40). Since the membrane serves as a solvent for the channel proteins, it is reasonable to assume that any parameter that influences the chemical and/or physical interaction between membrane and channel protein might also account for a differential activity of the WT channel and its mutants. Such membrane-based effects would remain undetected in the reduced bilayer recordings. We have seen in previous studies that the presence or absence of sterols or the concentration of anionic lipids is indeed able to affect unitary conductance and gating of the Kcv channel proteins (41).

An alternative and/or additional candidate for a hidden variable could be the number of channel proteins in the plasma membrane of yeast cells. This number is determined by a series of processes ranging from transcription over translation to the trafficking of the channel protein to its membrane of function. Because all channel mutants were properly synthesized and folded here in the in vitro translation system, we assume that the hidden variable is determined by downstream processes such as mutant-specific impacts on protein trafficking. This hypothesis is realistic because it was observed that minor mutations in Kcv-type channels can have profound impacts on intracellular protein trafficking (42,43,44).

In summary, our data confirm that the yeast complementation assay is a powerful and simple system for the screening of K^+^ channels. It allows for positive identification of functional K^+^ channels including even channels with low unitary conductance and/or very low open probability. Functional classification is, however, not complete for the full set of possible mutants; only about 50% of amino acids are correctly identified to be more or less active than the WT as observed from yeast growth. If the classification can be made more robust by including, for example, fluorescence-based measures of the plasma membrane density of the channel proteins, the yeast assay data could serve as a rapid surrogate quantity in future machine-learning approaches such as for the design of artificial K^+^ channels (15). Here, large data sets of protein variants with a confirmed and quantified activity are needed for training of these algorithms. In this kind of high-throughput design endeavor, at least all positively classified mutants need to be correctly predicted. Based on the present results, yeast growth alone cannot provide this classifier. Still, the positive correlation with at least more than 50% of possible mutants provides an optimistic perspective. In practice, as mentioned above, it will be necessary to augment the yeast data by other features such as an optical measure for the channel density in the membrane if this is indeed the most relevant hidden variable.

## Acknowledgments

Special thanks are extended to Prof. Adam Bertl for critical comments on yeast complementation assays. The work was supported by 10.13039/501100001659Deutsche Forschungsgemeinschaft (DFG, German Research Foundation) project ID 528562393-FIP 26 and grant TH558/34-1.

## Author contributions

K.K., M.C., M.G., and O.R.: acquisition, analysis, and interpretation of data; G.T., A.M., O.R., S.M.K., and L.E.S.: conception and design and analysis and interpretation of data; G.T. and O.R.: drafting of the article. All authors read, corrected, and approved the manuscript.

## Declaration of interests

The authors declare no competing interests.

## Supporting material

Document S1. Figures S1–S3 and Tables S1–S3

Document S2. Article plus supporting material
