# Supplementary material for: Yeast complementation assays provide limited information on functional features of K+ channels
Source: Biophys Rep (N Y). 2025 Mar 13;5(2):100206. doi: 10.1016/j.bpr.2025.100206 (PMC11985088; doi:10.1016/j.bpr.2025.100206)
Supplement: Document S1. Figures S1–S3 and Tables S1–S3 [file mmc1.pdf]

Biophysical Reports, Volume *5*

Supplemental information

Yeast complementation assays provide limited information on functional features of K^+ channels

Kerri Kukovetz, Matea Cartolano, Manuela Gebhardt, Lars E. Schumann, Stefan M. Kast, Anna Moroni, Gerhard Thiel, and Oliver Rauh

Supporting material

Yeast complementation assays provide limited information on functional features of K^+ channels

Kerri Kurkovetz[1], Matea Cartolano[1], Manuela Gebhardt[1], Lars E. Schumann[2], Stefan M. Kast[2], Anna Moroni[3], Gerhard Thiel[1,4], Oliver Rauh[1,4]

[1]*Department of Biology, TU Darmstadt, Darmstadt, Germany*

[2]*Department of Chemistry and Chemical Biology, TU Dortmund University, Dortmund, Germany*

[3]*Department of Biosciences and CNR IBF-Mi, Università degli Studi di Milano, Milano, Italy*

[4] *Institute for Functional Gene Analytics, Department of Natural Sciences, Bonn-Rhein-Sieg University of Applied Sciences, Rheinbach, Germany*

corresponding author: Oliver Rauh, Institute for Functional Gene Analytics, Department of Natural Sciences, Bonn-Rhein- Sieg University of Applied Sciences, Von-Liebig-Strasse 20, 53359 Rheinbach, Germany

e-mail: oliver.rauh@h-brs.de

TABLE S1

Mean data ± standard deviation (S.D.) for *N* independent recordings of measured current ($i_{meas\text{-}140}$) or true current ($i_{true\text{-}140}$) at −140 mV, the respective open probability at this voltage ($P_{O,-149}$) and the unitary conductance at positive voltages (g) for WT and mutant channels. Also reported are the mean increase of optical density OD_{600} (as relative units, r.u.) in N independent experiments generated by growth of yeast cells transfected with WT channel (L94), its mu tants (left panel) or empty vector (pYes) over 24 h (OD_{600}/24h) or 48 h (OD_{600}/48h) of incubation. The respective data are used for Figures 1C, 4A,B and 5 in main text.

single channel recordings										yeast complementation assay				
**94X**	**$i_{meas\text{-}140}$ [pA]**	**S.D.**	**$P_{O\text{-}140}$**	**S.D.**	**$i_{true\text{-}140}$ [pA]**	**S.D.**	**g [pS]**	**S.D.**	***N***	**OD_{600}/24h [r.u.]**	**S.D.**	**OD_{600}/48h [r.u.]**	**S.D.**	***N***
L94	-1.74	0.22	0.022	0.011	-26.3	1.36	183.43	9.04	8	11.98	3.058	19.021	3.242	3
L94R	-1.34	0.14	0.015	0.012	-6.2	1.45	40.39	19.38	3	3.011	1.198	11.568	1.221	3
L94K	-1.62	0.32	0.01	0.01	-12.9	0.64	83.11	20.26	3	16.71	5.170	26.148	8.932	3
L94H	-1.91	0.16	0.04	0.03	-15.5	2.26	103.74	13.61	3	20.53	7.543	32.928	8.115	3
L94G	-1.34	0.51	0.014	0.008	-20.2	3.29	147.53	26.53	4	1.963	0.091	7.409	0.731	3
L94C	-1.01	0.31	0.42	0.19	-22.1	2.66	155.82	13.02	6	2.175	0.990	13.122	5.248	3
L94P	-1.71	0.13	0.181	0.072	-17.4	0.62	157.92	9.66	8	12.98	5.016	20.725	2.065	3
L94Q	-2.99	2.45	0.036	0.022	-22.3	0.63	160.6	6.71	3	9.251	2.008	15.918	4.767	3
L94S	-1.31	0.14	0.009	0.007	-23.6	1.43	162.67	14.65	3	2.753	0.670	11.038	5.765	3
L94M	-1.69	0.27	0.0039	0.002	-22.1	2.66	164.53	19.96	3	15.98	1.536	28.437	4.939	3
L94N	-1.48	0.69	0.007	0.002	-23.7	2.00	167.6	11.32	3	1.799	0.291	3.901	1.056	3
L94A	-1.57	0.31	0.01	0.004	-23.7	0.41	169.23	7.46	3	5.211	0.926	13.987	2.188	3
L94I	-1.36	0.09	0.045	0.031	-24.8	2.39	170.64	11.82	4	2.362	0.570	19.936	4.577	3
L94W	-1.41	0.32	0.013	0.005	-24.4	1.88	171.88	22.51	4	1.878	0.297	15.725	3.329	3
L94Y	-1.53	0.8	0.0136	0.007	-20.0	8.71	172.81	8.41	4	15.58	3.126	25.58	8.323	3
L94V	-1.39	0.07	0.014	0.008	-24.5	0.63	173.22	8.44	4	13.90	4.381	21.348	0.644	3
L94F	-1.29	0.11	0.014	0.011	-25.4	0.62	182.59	6.79	6	17.94	3.189	29.388	6.342	3
L94D	-2.77	0.27	0.008	0.003	25.9	1.27	185.51	7.53	4	2.006	0.314	2.344	0.444	3
L94T	-1.92	0.18	0.0197	0.014	-27.3	1.89	188.36	16.89	3	10.84	3.394	17.249	2.692	3
L94E	-148	0.27	0.045	0.033	-26.4	1.94	192.13	14.39	7	2.574	0.994	8.664	2.508	3
pYes										1.557	0.047	1.455	0.136	3

TABLE S2

Correlation between OD_{600} after 24 h and 48 h and single channel parameters considering either absolute i_{meas} and i_{true} values or corresponding P_O values at ±140 mV or product of both parameters ($i \cdot P_O$).

**Single channel parameter / yeast growth**	**correlation coefficient**
$i_{meas,-140} \cdot P_{O,-140}$ /$OD_{600,24h}$	-0.12
$i_{meas,-140} \cdot P_{O,-140}$ /$OD_{600,48h}$	-0.05
$i_{meas,-140} \cdot P_{O,-140}$ /$OD_{600,24h}$	-0.17
$i_{meas,-140} \cdot P_{O,-140}$ /$OD_{600,48h}$	-0.07
$i_{meas,+140} \cdot P_{O,+140}$/$OD_{600,24h}$	0.20
$i_{meas,+140} \cdot P_{O,+140}$/$OD_{600,48h}$	-0.15
$P_{O,-140}$ /$OD_{600,24h}$	0.14
$P_{O,-140}$ /$OD_{600,48h}$	0.06
$i_{meas,-140}$ /$OD_{600,24h}$	-0.13
$i_{meas,-140}$ /$OD_{600,48h}$	-0.18
$i_{true,-140}$ /$OD_{600,24h}$	0.07
$i_{true,-140}$ /$OD_{600,48h}$	-0.13

TABLE S3

Pearson correlation coefficient and P-value of different data sets consisting of OD_{600} after 24 h and 48 h, respectively and absolute time-averaged current (abs. $i_{meas,-140} \cdot P_{O,-140}$), open probability ($P_{O,-140}$) at −140 mV, and absolute measured unitary channel current (abs. $i_{meas,-140}$).

**Data set**	**Pearson correlation coefficient**	***P*-value**
**abs. $i_{meas,-140} \cdot P_{O,-140}$ / rel. OD_{600}(48h)**	-0.0491926	0.836826
**$P_{O,-140}$ / rel. OD_{600}(48h)**	-0.0379763	0.873704
**abs. $i_{meas,-140}$ / rel. OD_{600}(48h)**	-0.165641	0.485229
**abs. $i_{meas,-140} \cdot P_{O,-140}$ / rel. OD_{600}(24h)**	-0.163205	0.491760
**$P_{O,-140}$ / rel. OD_{600}(24h)**	-0.152671	0.520492
**abs. $i_{meas,-140}$ / rel. OD_{600}(24h)**	-0.121687	0.609305

Figure S1

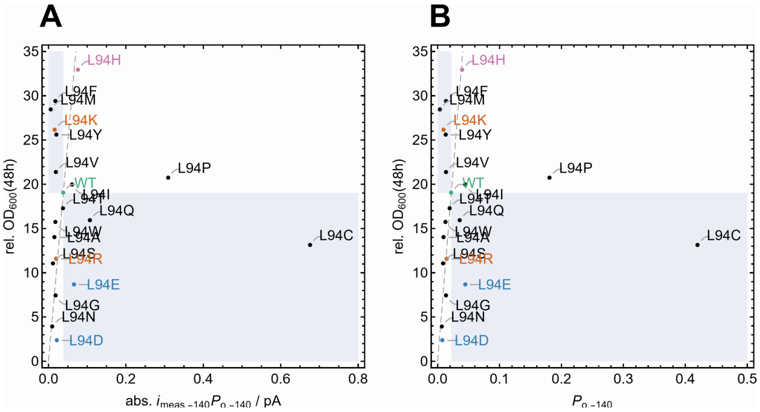

Fig. S1. Relative OD_{600} after 48 h against absolute time-averaged current (abs. $i_{meas,-140} \cdot P_{O,-140}$) (A) and open probability ($P_{O,-140}$) at −140 mV (B) as in Fig. 6 for all 20 amino acids in position 94.

Figure S2

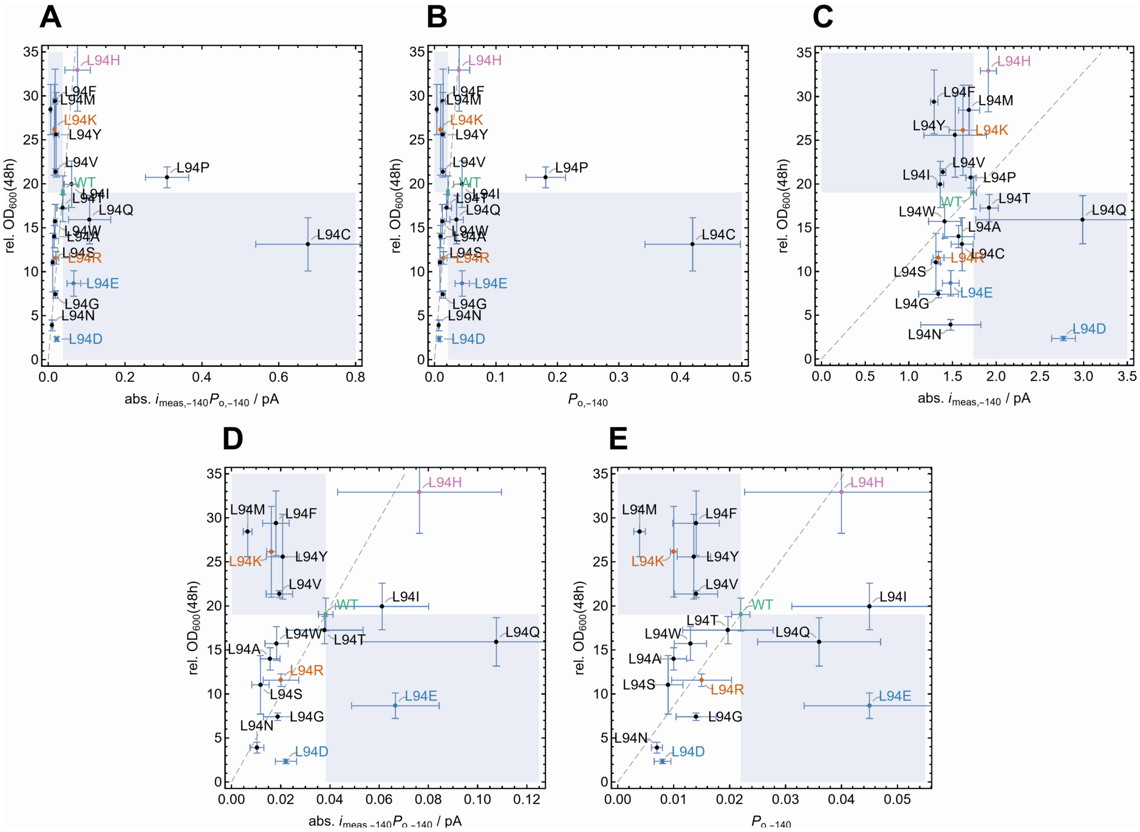

Fig. S2. (A-C): Mean relative OD_{600} ± standard error after 48 h against mean ± standard error of absolute time-averaged current (abs. $i_{meas,-140} \cdot P_{O,-140}$) (A), open probability ($P_{O,-140}$) at −140 mV (B), and absolute measured unitary channel current (abs. $i_{meas,-140}$) (C) for WT and L94X mutants; (D-E): corresponding panels A and B without L94P and L94C mutants.

Figure S3

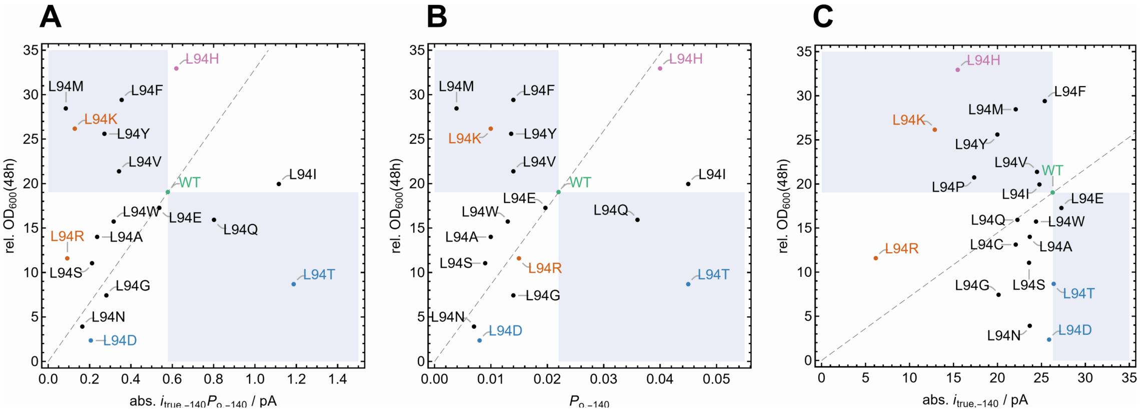

Fig. S3. Relative OD_{600} after 48 h against absolute time-averaged true current (abs. $i_{true,-140} \cdot P_{O,-140}$) (A), open probability ($P_{O,-140}$) at −140 mV (B), and absolute true channel current (abs. $i_{true,-140}$) (C) for WT and L94X mutants.
